# Tracing lineages to uncover neuronal identity

**DOI:** 10.1186/1741-7007-9-51

**Published:** 2011-07-19

**Authors:** Lia Panman, Thomas Perlmann

**Affiliations:** 1 Ludwig Institute for Cancer Research Ltd, Box 240, 171 77 Stockholm, Sweden; 2 Department of Cell and Molecular Biology, Karolinska Institute, 171 77 Stockholm, Sweden

## Abstract

Many previous studies have focused on understanding how midbrain dopamine neurons, which are implicated in many neurological conditions, are generated during embryogenesis. One of the remaining questions concerns how different dopamine neuron subtypes are specified. A recent paper in *Neural Development *has revealed features of a spatial and temporal lineage map that, together with other studies, begins to elucidate the developmental origin of distinct neuronal subtypes within the developing midbrain.

See research article http://www.neuraldevelopment.com/content/6/1/29

## Commentary

The complexity of the central nervous system (CNS), with hundreds of distinct neuronal cell types, emphasizes the importance of understanding the principles of how different types of neurons develop. During development, positional information is provided by extrinsic signaling factors. Signaling leads to the patterned expression of transcription factors within distinct domains of proliferating progenitor cells [1]. The fate of post-mitotic neurons - neurons that have exited the cell cycle and started to differentiate - is determined by combinations of transcription factors expressed in the progenitor cells. Post-mitotic differentiating neurons migrate, often to remote destinations, and become organized into distinct brain nuclei or cell layers. Neuronal fate is further specified and maintained by complexes of transcription factors expressed in the post-mitotic maturing neurons [2].

## Midbrain dopamine neuron development

Dopamine-secreting neurons (DA neurons) developing within the ventral midbrain are clinically important cells. Notably, the major motor symptoms in patients with Parkinson's disease are caused by DA neuron degeneration. Several other disorders, including schizophrenia and addiction, are associated with abnormal dopamine neurotransmission. Thus, the prospect of engineering DA neurons from stem cells has attracted considerable interest as it could provide opportunities for more advanced *in vitro *cellular models, allowing refined methods for drug development, and for cell replacement in patients with Parkinson's disease. However, stem-cell engineering requires detailed knowledge of how DA neurons are generated in normal embryogenesis, which in part explains a strong focus in previous studies on ventral midbrain development [3].

Although key signaling events and transcription factors important for the development of all DA neurons have been identified, much remains to be uncovered. Notably, the midbrain DA neurons can be subdivided into several anatomically and functionally distinct subgroups, but little is known about how these different types are specified. The rostrally, laterally located DA neurons of the substantia nigra pars compacta (SNc) are involved in motor control, and are the cells that mainly undergo degeneration in Parkinson's disease. Neurons of the ventral tegmental area (VTA) and retrorubal field are located at a caudal, medial position in the ventral midbrain and are part of the mesocorticolimbic system. Abnormal functioning of the limbic system is associated with psychiatric disorders, including addiction and schizophrenia. The different types of DA neurons resemble each other but their axon targets are distinct. Neurons of the SNc innervate the dorsal striatum while VTA neurons mainly innervate the more ventrally located nucleus accumbens and the prefrontal cortex. It remains unclear if these distinct DA neuron subtypes are developmentally specified at an early progenitor stage or if events in postmitotic neurons determine their subtype identity. Moreover, although recent studies have defined molecular markers that are uniquely expressed in distinct DA neuron progenitor domains, the lineage relationships between spatially distinct progenitors and different DA neuron subtypes remain unclear. A recent study published in *Neural Development *by Blaess *et al. *[4] provides new insights into these important questions.

## Lineage analysis of ventral midbrain DA neurons

After exiting the cell cycle, post-mitotic neurons migrate to distinct positions within the CNS. It is therefore difficult to predict the spatial and temporal origin of specific neuronal subtypes solely from analysis of gene expression in progenitor cells or from analysis of different mouse mutants. Blaess *et al. *[4] overcame this problem by using a technique called genetic inducible fate mapping (GIFM) to assess lineage relationships within the developing ventral midbrain. GIFM depends on using mice engineered to express an inducible Cre recombinase in specific progenitor cells. After crossing such mice with mice that express reporter genes - for example, enhanced yellow fluorescent protein - only after Cre-mediated recombination, specifically timed induction of the recombinase results in permanent marking of specific progenitor cells and their descendants. GIFM has been used successfully in many studies - for example, to determine the lineage of serotonergic progenitor cells [5], which led to new insights in rhombomere-specific contributions to distinct serotonergic nuclei. In addition, the timing of the establishment of a lineage-restriction boundary between the mesencephalon (the embryonic midbrain) and rhombomere 1 of the hindbrain has been determined using GIFM [6].

To study the origins of midbrain DA neurons, Blaess *et al. *used mouse strains in which Cre activity could be temporally controlled by tamoxifen administration, which switches on the Cre recombinase, to mark ventral midbrain progenitor cells expressing *sonic hedgehog *(*Shh*) and the Shh target gene *Gli1*. In normal development, expression of both genes is initiated in progenitor cells at the midbrain ventral midline and subsequently extends laterally, with *Gli1 *expression preceding that of *Shh*. A previous study by Joksimovic *et al. *[7] used an identical approach to mark *Shh*-expressing cells. These studies exploited the dynamic expression patterns of *Shh *[4,7] and *Gli1 *[4] combined with temporally controlled GIFM to follow the fate of distinct ventral midbrain progenitor pools.

Both Blaess *et al. *[4] and Joksimovic *et al. *[7] showed that distinct progenitor pools could be labeled using GIFM. Early tamoxifen treatment labeled a medial cell group (that is, cells nearest to the midline) whereas administration of tamoxifen at later time points labeled progressively more lateral progenitors. These results showed that the lateral expansion of *Shh *and *Gli1 *expression is due to induced expression in distinct progenitors rather than the lateral growth of early Shh-expressing progenitors.

Lineage maps were derived by analyzing the fate at embryonic day 18 [7] or postnatal days 21 to 30 [4] of *Shh*- and *Gli1*-expressing progenitors that had been marked at different times. Joksimovic *et al. *[7] concluded that the early *Shh*-expressing medial progenitors mainly give rise to neurons of the VTA, whereas Blaess *et al. *[4] found that these cells were preferentially generating neurons of the SNc. Unexpectedly, Blaess *et al. *also concluded that labeled lateral progenitor cells gave rise to astrocytes, whereas medial progenitor cells did not show any gliogenic potential [4]. Both studies consistently defined a population of more lateral and late *Shh*-expressing progenitors that preferentially give rise to neurons of the VTA but not the SNc. The discrepancies between the two studies can probably be explained by differences in the ways that the lineage maps were derived. For example, the two studies analyzed different markers and the fate-mapped cells were analyzed at different time-points. Regardless of these differences, however, both studies conclusively show that distinct *Shh*-expressing progenitor pools give rise to distinct types of DA neurons as defined at later embryonic or postnatal stages.

Although these studies provide useful results that begin to elucidate a lineage map of several different cell types of the ventral midbrain, they do not reveal the mechanisms by which distinct DA neuron subtypes are specified. While findings that distinct progenitor pools give rise to different types of DA neurons suggest that molecular differences in progenitors might determine the identity of the different subtypes, it is also possible that specification events take place at postmitotic stages. Indeed, the transcription factor Pitx3, which is expressed in postmitotic DA neurons, is important for the normal development of SNc but not VTA neurons [8]. Postmitotic expression of Otx2 was recently shown to be required for controlling the identity of Girk2-negative VTA neurons expressing low levels of the glycosylated dopamine transporter [9]. The neurotoxin MPTP (1-methyl-4-phenyl-1,2,3,6-tetrahydropyridine-HCl) selectively destroys dopaminergic neurons of the SNc and induces parkinsonian symptoms, while VTA neurons are relatively spared. One of the differentiated characteristics of VTA neurons conferred by Otx2 is higher resistance to MPTP.

**Figure 1:**
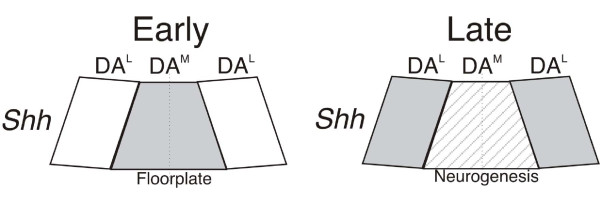
**Acquisition of neurogenic potential in midbrain floorplate cells**. From analysis of gene expression, the midbrain DA neuron progenitor domain can be subdivided into medial (DA^M^) and lateral (DA^L^) domains. Left, at early embryonic stages, medial DA progenitor cells resemble floorplate cells expressing high levels of *Shh *(pale gray). Right, downregulation of *Shh *(striped pale grey) in the medial progenitor domain at later stages in embryogenesis promotes neurogenesis from the medial progenitor domain.

Other studies have also begun to define molecular differences between medial and lateral DA neuron progenitor cells that may relate to DA neuron subtype specification. A remarkable feature of the development of DA neurons is that they can be generated at the midbrain ventral midline from floorplate cells with glia-like characteristics. In contrast, floorplate cells within the ventral midline of the hindbrain and spinal cord are unable to give rise to neurons. Thus, the midbrain floorplate cells are unique in their neurogenic capacity. The DA neuron progenitor zone can be molecularly defined as floorplate cells expressing the cell surface protein Corin and Shh, the latter only expressed at high levels at early stages of development, together with more lateral neuronal progenitors that lack floorplate characteristics and selectively express the signaling protein Wnt1 (Figure 1). Both Shh and Wnt1 are critical signaling molecules with important functions for many types of developing neurons, including DA neurons. Interestingly, downregulation of Shh expression within the ventral midline has been shown to be essential for the acquisition of neurogenic potential of floorplate cells [10] as shown by the expression of pan-neuronal transcription factor Ngn2. In addition, a recent study demonstrated that the characteristic DA neuron transcription factor Lmx1a is important for the appropriate onset of neurogenesis from the midbrain floorplate cells, whereas the related transcription factor Lmx1b is selectively required for the generation of DA neurons from more lateral Wnt1-expressing progenitors [11].

To further delineate the spatial and temporal contribution of distinct dopamine neuron progenitor populations, it would be interesting to determine the early and late fates of *Lmx1a*-, *Wnt1*- and *Corin*-expressing progenitor cells and subpopulations of these, using mouse strains with Cre recombinase under the control of various promoters. The study by Blaess *et al. *[4] clearly shows that the ability to mark different cell lineages in the ventral midbrain will be valuable for the generation of a more comprehensive lineage map that will also help to link molecular features and phenotypes in different mouse mutants to mechanisms that are essential for DA neuron subtype development. Importantly, such studies will also be instrumental in further efforts to effectively generate specific types of clinically important DA neurons from stem cells.
